# A strategy for developing new treatment paradigms for neuropsychiatric and neurocognitive symptoms in Alzheimer’s disease

**DOI:** 10.3389/fphar.2013.00047

**Published:** 2013-04-16

**Authors:** Hugo Geerts, Patrick Roberts, Athan Spiros, Robert Carr

**Affiliations:** ^1^In Silico BiosciencesBerwyn, PA, USA; ^2^Perelman School of Medicine, University of PennsylvaniaPhiladelphia, PA, USA; ^3^Oregon Health and Science UniversityPortland, OR, USA

**Keywords:** computer simulation, Alzheimer’s disease, apathy, cognitive disorders, drug discovery

## Abstract

Successful disease modifying drug development for Alzheimer’s disease (AD) has hit a roadblock with the recent failures of amyloid-based therapies, highlighting the translational disconnect between preclinical animal models and clinical outcome. Although disease modifying therapies are the Holy Grail to pursue, symptomatic therapies addressing cognitive and neuropsychiatric aspects of the disease are also extremely important for the quality of life of patients and caregivers. Despite the fact that neuropsychiatric problems in Alzheimer patients are the major driver for costs associated with institutionalization, no good preclinical animal models with predictive validity have been documented. We propose a combination of quantitative systems pharmacology (QSP), phenotypic screening and preclinical animal models as a novel strategy for addressing the bottleneck in both cognitive and neuropsychiatric drug discovery and development for AD. Preclinical animal models such as transgene rats documenting changes in neurotransmitters with tau and amyloid pathology will provide key information that together with human imaging, pathology and clinical data will inform the virtual patient model. In this way QSP modeling can partially overcome the translational disconnect and reduce the attrition of drug programs in the clinical setting. This approach is different from target driven drug discovery as it aims to restore emergent properties of the networks and therefore likely will identify multitarget drugs. We review examples on how this hybrid humanized QSP approach has been helpful in predicting clinical outcomes in schizophrenia treatment and cognitive impairment in AD and expand on how this strategy could be applied to neuropsychiatric symptoms in dementia. We believe such an innovative approach when used carefully could change the Research and Development paradigm for symptomatic treatment in AD.

## BACKGROUND

Development of successful medications for Alzheimer’s disease (AD) is extremely difficult; the Pharmaceutical Research and Manufacturing Association (PhRMA) estimates that over the last 12 years for every approved AD drug, 34 other drugs have failed [PhRMA (2012); resulting in an abominable success rate of less than 3%. All of these drugs passed preclinical animal tests successfully to the extent that companies were willing to invest large resources. There are many possible reasons for this translational disconnect, including different active metabolites, differences in affinities between the human and the rat targets, incomplete pathology in transgene animal models, the lack of genotypes that drive human variability and the inability to simulate comedication in preclinical animal models (Geerts, 2009; Markou et al., 2009)].

The search for disease modifying interventions is still the Holy Grail for AD (Holtzman et al., 2012). Most of the efforts have been focused on amyloid modulation, in particular gamma-secretase inhibition, passive vaccination or amyloid immunotherapy (Salomone et al., 2012) sofar without success (Castellani and Perry, 2012; Wolfe, 2012). Possible reasons for the failure include the choice of patient population and therefore new trial designs have shifted toward early and even presymptomatic AD (Ness et al., 2012). Another problem is the lack of validated biomarker that forces clinical trialists to use functional readouts or conversion rates, although efforts are ongoing for both biochemical and imaging biomarkers within the AD neuroimaging initiative (ADNI; Carrillo et al., 2012). Also the effect of reverting the underlying pathology might take a long time to manifest itself in functional readouts such as Alzheimer disease assessment scale-cognitive subscale (ADAS-Cog), making these trials prohibitively long and costly.

Other disease modifying approaches are actively investigated, for example antibodies against tau (Panza et al., 2012) and neuroinflammation (**Table 1**).

**Table 1:** List of failed therapeutic interventions in Alzheimer’s disease over the last 10 years, ordered according to the general mechanism. Many drugs failed in Phase II proof-of-concept, some failed in Phase III pivotal trials.

Drug	Mechanism	Clinical Phase
**Amyloid-beta modulation**
Bapineuzumab	Passive vaccination	PhIII
Solanezumab	Passive vaccination	PhIII
Semagecestat	Gamma-secretase inhibitor	PhII : irreversibly worsens cognition
avagacestat	Gamma-secretase inhibitor	PhII : irreversibly worsens cognition
ponezumab	Passive vaccination	PhII
Tramiprosate	Plaque destabilizer	PhIII
Scyllo-inositol	Block Abeta accumulation	PhII
tarenflurbil	Abeta lowering agent	PhIII
**Neuroprotective**
Latrepirdine (dimebon)	Mitochondrial stabilizer	PhIII
PF-04494700	RAGE inhibitor	PhII
cevimeline	Sensitizes neurons to growth factors	PhII
idebenone	Anti-oxydant	PhIII
buprofen, naproxen, rofecoxib	Anti-inflammatory	PhII
Atorvastatin, simvastatin	Cholesterol modulation	PhII
Leuprolide, neotrofin	Modulates growth factor	PhII
rosiglitazone	PPAR agonist	PhIII
Sabeluzole, T817-MA	Neuroprotectant	PhIII
**Symptomatic Treatment**
H3 antagonism, linopirdine, LU25-109, H4 agonism (PRX-03140), NS2330, ST101	Neurotransmitter modulator	PhII
Ispronicline, TC6683	A4b2 nAchR modulator	PhII
CX516	AMPAkine	PhII
Eptastigmine, huperzine, metrifonate, phenserine, physostigmine, propentofylline	AChE inhibitor	PhII
MEM1003	L-type Ca channel inhibitor	PhII
Milameline, sabcomeline, xanomeline, NGX267	Partial mAChR agonist	PhII
MKC-231	Improves cholinergic signalling	PhII
SGS-742	GABA-B antagonist	PhII
suritozole	Inverse GABA agonist	PhII
Nefiracetam, piracetam	Cognitive enhancer	PhII
Neramexane	NMDA antagonist	PhII

In contrast, a few symptomatic approaches have resulted in statistical significant and modest improvements on the functional ADAS-Cog scale. The 5-hydroxytryptamine-6 (5-HT6) antagonist SB 742457 has shown improvement in a 26 week trial (Maher-Edwards et al., 2010; Maher-Edwards et al., 2011) and the Envivo EVP6124 alpha7 nicotinic acetylcholine receptor (nAchR) modulator improved ADAS-Cog to the same extent as the currently approved acetylcholinesterase inhibitors (AChE-I; http://www.envivopharma.com/news-item.php?id=43).

Such a symptomatic effect is also important for off-target effects of possible disease modifying compounds; any off-target pharmacology that would reduce cognitive performance can lead to a failed clinical development program, even if the biochemical effect on the primary target is met. An example is dimebon, a mitochondrial stabilizer molecule (Eckert et al., 2012) which shows neuroprotection is preclinical models (Steele et al., 2012), but failed to show an effect in large phase III trials in AD (Bezprozvanny, 2010). Close study of its total pharmacology against the human receptors (Okun et al., 2010) reveals significant dopamine genotype-dependent anti-cognitive off-target effects that probably significantly reduces the clinical signal in ADAS-Cog (Geerts, 2012).

A similar situation can be found with regard to the neuropsychiatric symptoms in AD (Lesser and Hughes, 2006). Behavioral and psychological symptoms of dementia (BPSD), such as apathy, agitation, depression, and aggression are driving institutionalization and therefore contribute significantly to the overall cost of the disease. The currently approved AChE-I medications slightly improve the neuropsychiatric symptoms but it is unclear whether this is a specific effect or secondary to a cognitive improvement. In the absence of specific drugs for this indication, clinical trials are performed with drugs that have been approved for other indications. For instance, a 6 week clinical trial in 67 AD patients with apathy treated with methylphenidate (Mintzer et al., 2012) suggested a trend for improvement of 2.5 points on the AES (Apathy evaluation scale), and a significant improvement on clinical global impression of change (CGIC; 3.7 points) and on neuropsychiatric inventory (NPI).

The ideal treatment combination would be a multi-pharmacology profile that combines symptomatic improvement (for instance on the ADAS-Cog scale or NPI) with a disease modifying activity. This can substantially de-risk the whole research and development (R&D) project, as clinical trials can be executed using relatively short durations (26 weeks) and market approval can be sought based upon symptomatic improvement. Once on the market, long-term clinical trials or extensive studies using biomarkers can be performed to prove the disease modifying effect. This has the additional advantage that patent life can be used to its full extent.

This suggests that symptomatic treatment for cognitive or neuropsychiatric problems has an important place in the full Research and Development portfolio for AD.

## PRECLINICAL MODELS FOR COGNITIVE ENHANCERS

Traditional cognitive tests such as object recognition, T-maze, set shifting, and Morris water maze have been performed for a long time leading to an impressive literature and a profound understanding of the (rodent) brain neurophysiology that leads to the observed outcomes. When coupled with animal models that reflect part of Alzheimer pathology, reversal of a cognitive deficit can be demonstrated with many candidate drugs. Many of these same drugs do not work in the clinical setting (Thomsen et al., 2010). Possible solutions discussed at a recent workshop, “Improving the utility and translation of animal models for nervous system disorders”, on this topic organized by the Institute of Medicine (http://www.nap.edu/catalog.php?record_id=13530) include bringing in aspects of human clinical trial management, such as power calculations, standard operating procedures, blinding of observers and comedication into the preclinical world in addition to a more organic integration of quantitative systems pharmacology. 

Another issue is the alignment of preclinical test readouts and human clinical scales. In the field of cognitive impairment associated with schizophrenia (CIAS), The National Institutes of Health (NIH)-sponsored meetings between Food and Drug Administration (FDA), academia and industry led to the adoption of the clinical Matrics scale and the identification of preclinical animal readouts that would correspond to the different clinical dimensions (for a review see Young et al., 2009). The recently launched NewMeds Initiative supports the validation of more relevant readouts in rodent preclinical animal models using touch screen technology (Bartko et al., 2011) in an attempt to bridge this translational disconnect.

While many of these ideas will improve the reproducibility of preclinical animal test within different laboratories, it is unclear whether this will lead to improved success in the clinical development phase. There are still fundamental differences linked to the choice of rodents as preclinical animal models. For instance in the field of cognition, there is increasing evidence that the excitatory-inhibitory (e-i) balance in cortical and hippocampal networks is fundamentally different between primates and rodents (Povysheva et al., 2008), in that monkey basket interneuron cells have a higher input resistance and a lower firing threshold, and they generated more spikes at near-threshold current intensities. In the rodent, gamma-aminobutyric acid (GABA)ergic neurons represent only about 15% of all cortical neurons (Beaulieu, 1993), while in the macaque monkey they represent up to 20% in visual cortex and 25% in other cortical areas (Hendry et al., 1987; Beaulieu et al., 1992). Different interneuron subtypes with short spike duration are found in the primate cortex, which is not typical for rodent adapting cells (Zaitsev et al., 2009). Furthermore, the developmental shift in GABA(A) receptor alpha subunit expression continues through adolescence in primate cortex, but not in rodents, suggesting species difference kinetics of GABA neurotransmission (Hashimoto et al., 2009).

## PRECLINICAL MODELS FOR NEUROPSYCHIATRIC SYMPTOMS IN ALZHEIMER’S DISEASE

While there are many preclinical models and readouts for cognition, there are almost no animal models that are typically developed for neuropsychiatric behavioral symptoms in dementia. Neuropathological studies indeed suggest a profound and mostly tangle-driven locus coeruleus (LC) pathology early on in the disease (Palmer and DeKosky, 1993; McMillan et al., 2011) and additional LC pathology through treatment of mice with N-(2-chloroethyl)-N-ethyl-bromobenzylamine (DSP4) can exacerbate olfactory dysfunction in traditional amyloid precursor protein (APP) transgene mice (Rey et al., 2012).

The dorsal raphe (DR) also shows neurofibrillary tangle pathology (Grinberg et al., 2009) at an early stage where entorhinal cortex pathology is not yet fully established, although an imaging study in patients suggest that postsynaptic neuronal cell loss occurs before loss of serotonergic projections (Marner et al., 2012). This is in line with the observation that DR pathology is observed more in tauopathy transgene mice than in amyloid-related transgene mice; for instance DR pathology in the P301L tau transgene mice leads to serotonin metabolism and breathing activity changes (Menuet et al., 2011).

This suggests that brainstem pathology in serotonin and noradrenergic nuclei, in principle can be observed in specific tau transgene animal models and further exacerbated either by neurochemical or behavioral means For instance, chronic stress, induced by isolation and chronic restraint can lead to additionally mono-amine dysfunction (Jeong et al., 2006; Cuadrado-Tejedor et al., 2012). However, most behavioral readouts were focused on cognitive impairment rather than mood states. In addition, due to the emphasis on amyloid-based models, not many studies have been performed on relevant behavioral readouts in tau transgene mice.

Possible readouts in preclinical animal models include the resident-intruder test for aggression; locomotor hyperactivity; forced swim test and tail suspension test for depression and saccharine preference or progressive ratio for reinforcement for apathy.

This overview suggests that mono-amine dysfunction is present in the early stages of the disease, is dominantly tau pathology driven and can affect the behavioral problems associated with dementia. This would suggest that in order to develop an R&D strategy with preclinical animal models requires a shift from amyloid-based models to tau-based models and from purely cognitive readouts to readouts that probe mood dysfunction. However some transgene APP mouse models also show behavioural disturbances, such as the 3xTg-AD model (Sterniczuk et al., 2010) that might be an interesting animal model as it combines both APP and tau pathology. 

However, even when achieving face-validity at the level of neuropathology in animal models, possible drug discovery could still be substantially impaired because of the species differences in receptor distribution and neuromodulator synapse physiology. For instance the striatal dopaminergic synapse is substantially different between rodent and primate (Spiros et al., 2010), to the extent that this led to the clinical failure of the partial D_2_ agonist bifeprunox, despite the fact that this compound achieved a higher efficacy in preclinical rodent-based animal models than the similar successful drug aripiprazole. Also, the distribution of the 5-HT_3_R, a receptor involved in negative symptoms in schizophrenia (Akhondzadeh et al., 2009) and in attention deficit (Lennertz et al., 2010) is fundamentally different between mice and men (Hewlett et al., 1998; Marazziti et al., 2001), suggesting that changes in serotonin tone can lead to very different behavioral outcome.

## QUANTITATIVE SYSTEMS PHARMACOLOGY

The previous discussion suggests that improved translation can be achieved by developing a hybrid computer-based model that combines the best of preclinical neurophysiology with actual human imaging, postmortem, and clinical data. Such an approach is based upon a biophysically realistic computer model of neuronal networks that takes into account proper target engagement of central nervous system (CNS) active drugs and well documented effect of receptor activation level changes on ion-channel conductances that ultimately modulate neuronal excitability and timing of action potential generation (Geerts, 2012). In addition calibration of the biological coupling parameters using the correlation between clinical outcomes and the outcomes of the same drugs in the computer platform ensures a tight clinical association between model output and actual anticipated clinical result.

Details of the platform have been described elsewhere (Spiros et al., 2010; Roberts et al., 2012b). With regard to cognitive disorders, we have used this platform to explain the differential clinical effect of memantine and apolipoprotein E (APOE) on ADAS-Cog in Alzheimer patients (Roberts et al., 2012b). The platform also predicted correctly a completely unexpected phase I outcome in humans with the lowest dose of a novel symptomatic compound on scopolamine-induced deficit that was different from what preclinical animal models suggested (Nicholas et al., 2012). This was caused by a different pharmacology for the human vs. the rodent targets and the human-specific dynamics of the neuromodulatory transmitter. The higher dose was less accurately predicted; however, the outcome was substantially better in a subsequent iteration of the model using the feedback from this trial.

We implemented the human-specific effects of the catechol-O-methyl transferase (COMT) Val 158Met genotype (Spiros et al., 2011) based upon imaging studies in healthy volunteers with the D_1_R specific radiotracer NNC-112 (Slifstein et al., 2008). This helped to identify dimebon’s off-target effect at the D_1_R as a strong driver of cognitive effects of dimebon in AD and to show that this was further modulated by strong COMTVal158Met genotype effect on the outcome, possibly explaining one of the reasons for its failure (Geerts, 2012).

With regard to psychiatric symptoms, the model correctly and blindly predicted a phase II outcome in the positive and negative symptoms in schizophrenia (PANSS) total scale for a novel drug in schizophrenia (Geerts et al., 2012). We predicted an unexpected motor side-effect that was not observed in preclinical animal models, but later confirmed in the clinical trial. In the same paper, we failed to accurately predict the absolute clinical outcome of a second drug, however, we correctly predicted the relative clinical performance compared to olanzapine. The platform also identified the specific species difference of striatal dopaminergic synapses that led to the unexpected clinical failure of bifeprunox, despite outperforming aripiprazole in preclinical animal tests (Spiros et al., 2010).

This suggests that using the hybrid approach where preclinical neuropathology derived from specific animal models in behavioral disturbance is combined with a quantitative systems pharmacology (QSP) model has the capacity to improve the clinical predictability.

## STRATEGY FOR A NOVEL PRECLINICAL MODEL OF BEHAVIORAL PROBLEMS ASSOCIATED WITH DEMENTIA

We propose to combine preclinical neurophysiology, human neuropathology, and human clinical data in a mechanism-based computer model for developing new drugs for the BPSD. We outline a strategy aimed at (1) documenting better the neuropathological changes in modulatory neurotransmitters in AD by studying the effects of AD pathology on LC, DR, and ventral tegmentum area, preferably in transgene rats, (2) comparison with the neuropathology observed in human patients, especially with regard to functional connections between different brain regions, (3) build a computer model of these interactions based upon the human imaging template but informed by the neurophysiological changes in cellular excitability observed in transgene rats and (4) identify and calibrate emergent properties of the network model by simulating various clinical trials with CNS psycho-active drugs and compare this to the clinical outcomes, and (5) use this virtual patient model to perform *in silico* screening.

Below, we expand on different steps.

## DERIVING INFORMATION FROM PRECLINICAL ANIMAL MODELS

Preclinical data from transgene rats (Liu et al., 2008) are preferred because of LC and DR size considerations and because electrophysiology of the rat striatum can be done in awake animals (Li et al., 2012). Lentivirus-mediated gene transfer with mutant tau (Khandelwal et al., 2012) can enhance the pathology in the LC and DR. The striatal dopamine system is an important part of the reward circuit involved in mood regulation, while noradrenergic amygdala-related electrophysiology changes can be detected. At the same time, *in vivo* electrophysiology can be used to document the downstream functional consequences of this pathology. We believe electrophysiology readouts are much closer to the behavioral readouts in patients and animals than biochemical changes and therefore are better suited for translation into the clinical setting.

## HUMAN IMAGING, PATHOLOGY, AND GENETICS

A large number of imaging studies have been performed in the framework of ADNI. They include morphometric, blood-oxygen level dependent functional magnetic resonance imaging (BOLD fMRI), functional connectivity and positron emission tomography (PET) imaging modalities (Carrillo et al., 2012). Progress has been made to delineate the functional brain networks in various psychiatric conditions (Williamson and Allman, 2012), identifying emotional encoding, representational brain and directed effort networks that are differentially affected in schizophrenia and mood disorders and that can function as a starting point for complex model implementation. In addition some genetic markers have been found to associate with specific domains of behavioral disturbance that can further inform the computer model (Proitsi et al., 2012).

As an example, human imaging studies (Kuhn and Gallinat, 2011) can be used for implementation of a model for negative symptoms (Geerts et al., 2013). Indeed, in schizophrenia patients, imaging studies suggest a ventromedial prefrontal cortex (vmPFC) BOLD fMRI activity that is inversely related to the degree of anhedonia (Harvey et al., 2010; Lee et al., 2012) associated with a corresponding lower ventral striatum activation (Juckel et al., 2006). This allows defining the pathological changes and the relation between brain regional activity and clinical scores on anhedonia scales. Using these imaging data together with neurophysiology data on glycine dynamics, a model has been constructed that explores the non-linear dose-response of glutamatergic interventions, such as Glycine transporter-1 inhibitor in negative symptoms in schizophrenia (Geerts et al., 2013).

## CORTICAL-SUBCORTICAL COMPUTER MODEL

The model of subcortical circuitry consists of three components: striatum, striatum mediale-globus pallidus (STM-GP) circuitry, and thalamus (**Figure 2**). The striatum is based on a previous model extended from [58] where we have added receptor effects for pharmaceutical Research and Development. The STM-GP circuitry will consist of two segments of the GPe and GPi and the subthalamic nucleus (STN; Rubin and Terman, 2004). An existing computational thalamus model (Bazhenov et al., 1998) is extended with receptor effects for pharmaceutical research.

### Striatum component

**FIGURE 1:**
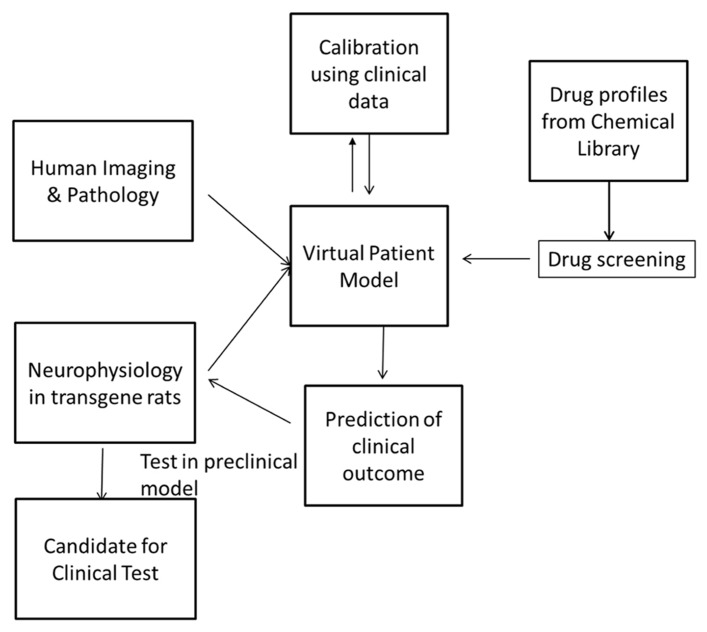
**Screening can be performed by simulating the pharmacology of existing drugs from chemical libraries such as the Prestwick library and identifying these with the greatest beneficial effect on the clinical outcome**. These can then be tested in the preclinical animal model before they are tested in the clinic. In this way, the drug discovery and development process can be highly streamlined and made more efficient.

The striatum model simulates the processing capacity of generic medium spiny neurons (MSN) in the ventral striatum or nucleus accumbens. The model is described in detail elsewhere (Spiros et al., 2011). The model for the direct and indirect pathways is based on the Rubin and Terman (2004) model of STN, GPi, and GPe. Each nucleus contains 16 of neurons with the following membrane currents: sodium current (Na), delayed rectifier potassium current (Kdr), T-type calcium current (CaT), L-type calcium current (CaL), and a leak current (**Figure 1**). Receptor modulation of these neurons by dopamine, serotonin, acetylcholine, etc., will be obtained from the literature.

The STN, GPe, and GPi population is divided in two groups of eight cells each. Each GPe cell receives inhibitory input from 3 other GPe cells and also receives excitatory input from three STN cells while Each STN cell receives inhibitory input from 3 GPe cells. Each GPi cell is coupled to an inhibitory input from a GPe neuron and excitatory input from a STN neuron. The thalamocortical (TC) cells of the thalamus all receive inhibitory input from the GPi cells. Synaptic couplings are similar to the ones implemented in the working model.

### Thalamus component

**FIGURE 2:**
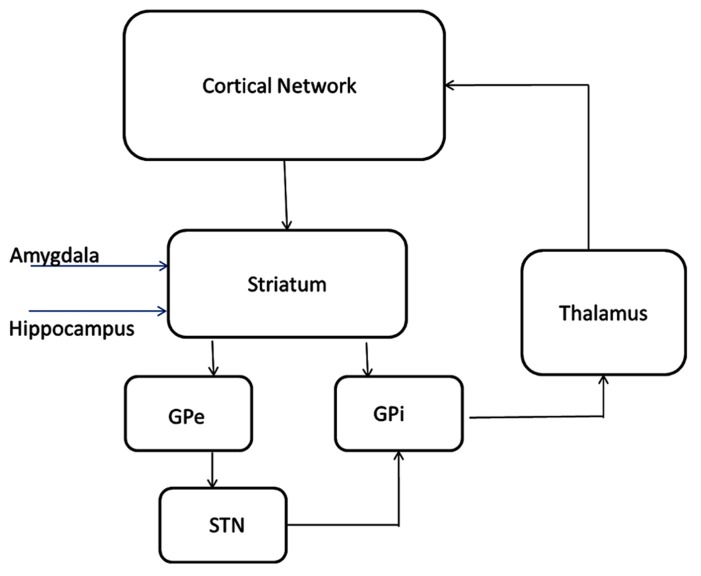
**Circuitry and receptor effects for the proposed cortical-basal ganglia-thalamic model**. A working memory subcircuit (Durstewitz et al., 2000) maintains a burst of activity representing working memory span. Two types of medium spiny neurons in the striatum (Gruber et al., 2003), D1 and D2 project inhibitory (-) synapses to GPi (direct pathway) and GPe (indirect pathway) respectively. The GPe neurons are reciprocally coupled to themselves and the subthalamic nucleus (STN; Rubin and Terman, 2004; Pirini et al., 2009). The STN projects excitatory (+) synapses to the GPi and the GPi projects inhibitory connections to the thalamus (Bazhenov et al., 1998). Thalamocortical (TC) neurons excite reticular (Re) neurons that reciprocally inhibit TC. Sensory input excites TC and TC projects to the cortex. All cell types receive background excitatory and inhibitory fluctuating inputs that represent random synaptic activity. White rectangles represent membrane currents and colored ovals represent receptor types.

The model is based on the circuitry and cellular properties of the model reported by (Bazhenov et al., 1998) with TC and reticular (Re) neuronal cell types. The TC neurons are excitatory, glutamatergic relay neurons that pass sensory information to the cortex, while the Re neurons are inhibitory, GABAergic feedback neurons that receive inputs from, and inhibit, TC neurons (**Figure 2**). The synaptic interaction between these neuronal types, and their intrinsic membrane properties, leads to oscillations and suppression of multiple input signals.

The spiking properties of TC neurons is caused (Traub et al., 1991) by a fast sodium channel, a fast potassium channel, a low-threshold Ca channel, a hyperpolarization-activated cation channel, a potassium A channel, and a potassium leak channel. To model an RE cell, a fast sodium channel, a fast potassium channel, a low-threshold Ca channel, and a potassium leak channel are included. Background noise caused by synaptic bombardment by excitatory and inhibitory neurons is represented by a stochastic model (Destexhe et al., 2001) containing two fluctuating conductances. Muscarinic M_2 _receptor activation increases the maximum conductance of h channels in TC cells and the maximum conductance of leak channels in Re cells. Serotonergic 5-HT_2_A receptor activation increases the maximum conductance of Leak channels in Re cells.

## CORTICAL NETWORK MODEL FOR COGNITIVE ENHANCEMENT

The cortical network model for cognitive function is described in detail elsewhere (Roberts et al., 2012b). Basically, we extended a biophysically realistic model of a network comprised of four-compartment pyramidal cells and two-compartment GABA interneurons (Durstewitz et al., 2000; DeFelipe, 2002) with the receptor physiology of 18 different dopaminergic, serotonergic, noradrenergic, and cholinergic receptors to a system of 80 four-compartment pyramidal cells and 40 two-compartment interneurons (**Figure 2**). An mGluR5-dependent *delayed afterdepolarization *current that can increase the spiking rate of pyramidal cells for several seconds was implemented as an alpha function in the**model with a time constant similar to the observation in (Sidiropoulou et al., 2009). In contrast to the original network, 40% of the interneurons do not synapse with pyramidal cells but form a small local recurrent network, based on insights from the relative number of pyramidal cells and interneurons (DeFelipe, 2002).

A stimulus is initiated by injecting a current at *t* = 2000 ms which starts the firing of the target pyramidal cells. Without further stimuli, this synchronized firing pattern goes on for a certain amount of time before it gets degraded by the background noise and the interference of the distractor neurons. This time span, called the working memory span, is usually in the range of 4–10 s and corresponds to the time a certain pattern is held in working memory (Levy and Goldman-Rakic, 2000).

A time span, called working memory span, is defined as the time a synchronous firing in the neurons that are stimulated is sustained without further stimulation. We first divide the time axis in bins of 200 ms and count the number of neurons firing in that time window and determine the time points where this number exceeds M/2, where M is the number of neurons stimulated at *t* = 2000 ms. The time difference between these two transition points is the memory span.

In general, we assume a linear normalized relationship between receptor activation and biological effect on physiological responses such as $X_{\text{Y}}^{\text{eff}}=\frac{X_{\text{Y}}^{\text{A}}-X_{\text{Y}}^{\text{C}}}{X_{\text{Y}}^{\text{C}}};$ where $X_{\text{Y}}^{\text{A}}$ and $X_{\text{Y}}^{\text{C}}$ are the actual activation levels of receptor X subtype Y (for instance 5-HT_6_) after treatment (A) and the untreated (healthy) control levels (C).

The parameters coupling the documented intracellular processes with these receptors are further calibrated using the correlation between the effect of therapeutic interventions in the network and their clinical working memory performance on the ADAS-Cog scale in Alzheimer’s patients (see below).

Alzheimer’s disease pathology is implemented as a loss of cortical neurons (Ball, 1977) at a rate *Neuron_Loss* (%/week) and synapses from pyramidal neurons (Masliah et al., 1989) with a rate *Synapse_Loss* (%/week). Both excitatory-excitatory (e-e) and e-i are eliminated at the same rate, but because there is an additional pyramidal cell loss, e-e synapses tend to decrease faster.

Reduced cholinergic tone (Davies and Maloney, 1976) is implemented as a free parameter *ACh_decrease* on all cholinergic receptors (M_1_ and M_2_ mAChR, and α_7_ and α4β2 nAChR). In addition we introduce a placebo effect at week 12 using an increase in general cortical dopaminergic tone (Boileau et al., 2007). The size of the DA placebo effect is calibrated as well.

LC and DR pathology will be implemented as derived from human neuropathology data. Basically the decrease in norepinephrine (NE) tone as a consequence of noradrenergic tau pathology will be reduced by a compensatory mechanism (McMillan et al., 2011), that however, will get smaller as the disease progresses, while DR pathology will be implemented as a decrease in 5-HT projection density as determined by 2-dimethylaminomethyl-phenylsulfanylbenzonitrile (DASB) imaging (Marner et al., 2012).

## CALIBRATION OF THE NETWORK WITH CLINICAL DATA

For the calibration of the AD cortical network, we collected the publicly available data from clinical trials in mild-to-moderate AD patients using ADAS-Cog clinical scales (Ito et al., 2010), together with reported clinical data on 5-HT6 antagonists (Maher-Edwards et al., 2011) and placebo data at 78 weeks from tarenflurbil and tramiprosate trials (Gauthier et al., 2009; Green et al., 2009; Schneider and Sano, 2009). All together 28 different clinical interventions over four time points (12, 26, 52, and 78 weeks), four drugs (donepezil, galantamine, rivastigmine, and SB742457) with up to three doses are used. The correlation between model outcome and clinical results is about 0.76 (Roberts et al., 2012a,b), suggesting that the model outcome is able to relatively accurately predict the functional outcome of new therapeutic agents on the ADAS-Cog scales.

As for clinical data on neuropsychiatric symptoms in AD, published publicly available data on the effect of CNS active drugs on the NPI can be used to calibrate different subdomains of behavioral disturbance. Many antipsychotics and antidepressants have been tested in this patient population. Additional clinical scales include the behavioral pathology in AD rating scale (BEHAVE-AD), the Cohen-Mansfield Agitation Inventory (CMAI; De Deyn and Wirshing, 2001), while extrapyramidal symptoms can be assessed using the extrapyramidal symptom rating scale.

## DISCUSSION

This position paper documents the need for drug discovery and development focused on neuropsychiatric symptoms in AD and outlines a strategy for a rational approach. The need for innovative therapies that substantially address the behavioral problems associated with dementia is driven by the fact that these symptoms drive a large part of the economic costs associated with institutionalization.

Target driven discovery paradigms in general have resulted in disappointing clinical outcomes; in fact a retrospective study (Swinney and Anthony, 2011) of FDA-approved medications over the last 10 years and over all indications has found that a majority of successful drugs have been identified using phenotypic assays, despite a tremendous investment in reductionist genomic-based approaches. While this study does not explicitly mention CNS indications, it is safe to assume that in these indcations, target-derived approaches are even less successful. However, a phenotypic assay for CNS indications likely has a very low throughput and includes rodent or non-human primate models. As a notable exception, the company psychogenics has optimized a relatively high-throughput behavioral screening systems (Brunner et al., 2002) that is fully automated. However, the limited translationability of rodent models for psychiatric or neurological indications seriously hampers the usefulness of these phenotypic models.

The humanized QSP approach offers the possibility of circumventing these limitations because it is a hybrid platform that is heavily populated with human clinical data, calibrated and validated with clinical outcome tests and as such can provide a platform with more translational predictability at least for symptomatic functional interventions, such as neuropsychiatric symptoms in AD. In addition, the feedback for every prediction the model makes, can be used to improve the platform in a learn-and –confirm cycle (Sheiner, 1997).

Once validated, the QSP model can be used to either screen the effect of the pharmacology of marketed drugs in a repurposing strategy. The platform can also screen *in silico* the pharmacology of large numbers of compounds in the libraries of pharmaceutical companies once their affinities against human receptors have been documented. Almost all drugs that have entered clinical trials for many indications and all compounds that have been tested for CNS indications, probably have their pharmacology against human receptors tested.

Alternatively the model can be reverse engineered to identify the optimal profile of a new compound that would improve the clinical outcome substantially and a drug discovery program can then be initiated, hopefully resulting in a multi-pharmacology drug that can be developed in the clinic. A similar project is currently ongoing in oncology (Merrimack, 2012) here a novel drug target was identified using computational modeling and a drug discovery program driven by this QSP approach resulted in a new drug that is currently being tested in the clinic. This is a very rational way to develop new drugs that would address emergent phenotypic changes rather than being focused on a single-target.

Additionally this platform can be used to evaluate the off-target effects of disease modifying small molecules on neurocognitive and neuropsychiatric symptoms. In fact, because functional clinical scales are the primary end-parameter for any clinical trial, it would be a pity if a drug that really affects the primary cause of the disease would fail in the clinic, because of off-target pharmacology that would significantly reduce clinical readouts. As an example, dimebon’s failure in AD can partially be attributed to its off-target effects at the dopamine D_1_ and the histamine H_1_ receptor (Geerts, 2012). The QSP platform for cognitive symptoms in AD is offered as a research service (ADDF, 2012; Geerts, 2012) to the Alzheimer Drug Discovery Foundation network.

In summary this report describes a novel strategy to screen new drugs for the challenging indication of behavioral disturbances associated with senile dementia and for symptomatic improvement of cognitive symptoms.

## Conflict of Interest Statement

All authors are employees of In Silico Biosciences.
